# Editorial for special issue: Metabolomics in India

**DOI:** 10.1002/ansa.202100064

**Published:** 2021-12-23

**Authors:** Biswapriya B. Misra, Dietrich A. Volmer

**Affiliations:** ^1^ Independent Researcher New Delhi India; ^2^ Humboldt Universität zu Berlin Berlin Germany

The explosive growth of metabolomics has greatly reinvigorated interest in metabolism from a systems biology perspective. With a growing knowledge‐based economy in India, the field of metabolomics has seen impressive growth in the past decade, thanks to the well‐educated and skilled scientists of this country.

In order to capture the progress and advances in metabolomics, we decided on a special issue in early 2020 for this new journal, Analytical Science Advances (ASA), and we could think of no better topic than “Metabolomics in India,” to provide the Indian metabolomics community with a platform to showcase its exciting new developments. We invited authors to submit articles that focused on topics from analytical mass spectrometry to omics technologies and received many exciting articles. After rigorous peer‐review over the past 12 months during the COVID‐19 pandemic, the process resulted in the eventual acceptance of 12 articles. The original research articles used either nuclear magnetic resonance (NMR), LC‐MS/MS, or GC‐MS as tools for generating metabolomics scale data.

Mishra et al^1^ demonstrated the use of UHPLC‐ESI‐MS/MS in bioprospection of stem bark materials of *Betula utilis* collected from various geographical regions in India, leading to identification of 10 bioactive triterpenoids, phenolics, and flavonoids using a targeted multiple reaction monitoring (MRM) approach. Such validated methods with respect to linearity, intra‐ and interday precision, and accuracy will help the field move forward in areas of confident quantification of bioactive substances from phytochemical resources.

Rendedula et al^2^ demonstrated the resolving power and sensitivity of solid‐phase extraction (SPE) ultra‐high performance liquid chromatograph (UHPLC) hyphenated to quadrupole hybrid Orbitrap mass spectrometry (Q‐Orbitrap‐MS) in capturing 26 phthalates, pharmaceuticals, and personal care products (PPPCPs) in river water samples from an Indian river, the Ganges.

Colvin et al^3^ adopted a proteomics and metabolomics guided approach to demonstrate their utility in mosquito larvicidal toxicity in Bacillus sp. Isolated from the mid‐gut of *Culex quinquefasciatus* larvae. They reported differential toxicities of the four identified bacterial strains to specific metabolites and proteins occurring in the pathogens, indicating the importance of microbial ID and profiling efforts in infectious disease research.

Guleria et al^4^ used an NMR‐based untargeted metabolomics effort in serum and muscle tissues for diagnosis and activity assessment of idiopathic inflammatory myopathies. Metabolic profiles of sera (N = 99) and muscle (N = 21) from patients with idiopathic inflammatory myopathies were compared with healthy control (HC) samples (N = 75 for serum and N = 12 for muscle tissues) employing 800 MHz NMR spectroscopy. The patient sera showed a visible shift to anaerobic metabolism (increased lactate, low glucose), oxidative defect (high phenylalanine/tyrosine), whereas three metabolites (isopropanol, succinate, and glycine) were distinctive in muscle tissue metabolomics. The authors demonstrate the power of untargeted NMR metabolomics in its ability to differentiate active from inactive myositis, where conventional markers fail, and in aiding in decision making for continued medication approaches, that is, immune‐suppressant continuation in this case.

In another NMR‐based targeted metabolomics effort by Kumar et al,^5^ serum metabolic profiles of 98 takayasu arteritis (TA)‐patients and 77 normal controls (NC) samples were measured using a high‐resolution 800 MHz NMR spectrometer employing standard 1D‐1H‐CPMG NMR experiments, showing that histidine levels were significantly decreased in active TA patients (23.90; IQR:16.10). The authors noted that circulatory levels of histidine in patients can serve as a surrogate marker for improving diagnostic screening in active and inactive TA patients.

Another NMR‐based targeted metabolomics study by Arya et al^6^ investigated the circulatory levels of serine and glycine in the sera of 40 ACLF patients and 49 normal controls (NC) subject using 1D 1H CPMG NMR spectra that provided insights into the potential mechanisms of acute‐on‐Chronic liver failure (ACLF) in cirrhotic patients. The authors showed that the circulating levels of serine and glycine were significantly decreased in ACLF patients (Ser = 23.06 ± 1.67 and Gly = 83.11 ± 7.52) compared to NC subjects (Ser = 55.61 ± 2.28 and Gly = 156.9 ± 7.16) with *p*‐value < 0.0001, indicative of depressed immune system and mitochondrial dysfunction.

Ganneru et al^7^ demonstrated the use of GC‐MS based metabolomics in revealing metabolic perturbations in *Carica papaya* Linn. (papaya), which has been ripened either by the ripening practice (room temperature process as control) and/or ripening agents (calcium carbide and ethylene). The authors recorded significant alternations in 13 metabolites mainly sugars, amino acids, fatty acids, and organic acids as well as disturbances in five metabolic pathways due to different ripening practice/agents.

This Special issue also received four review manuscripts. Chandran et al's review^8^ underscored the role of quantitative metabolomics in early diagnosis of inborn errors of metabolism (IEM) in India. Given that the predictive cumulative incidence of IEMs is said to be ∼1:800 newborns in Indian population, applying LC‐MS/MS‐based screens using targeted and untargeted approaches new born screening for IEM opens up immense possibilities for the Indian healthcare system. Of note, we commend the impressive set of infographics and images created by the authors in this succinct review.

In another review on the application of magnetic resonance spectroscopy (MRS) in breast cancer, Sharma and Jagannathan^9^ compare the technology with mass spectrometry and demonstrate a handful of applications in biomedical research, which goes on to show its enormous potential in capturing the underlying metabolic reprogramming in breast cancer cells, in unravelling molecular biomarkers, and applications in in vitro and ex vivo studies.

A review by Puri et al^10^ highlights the role of diverse spectroscopic techniques in discovery of phytochemicals from medicinal plants. The manuscript introduces targeted and untargeted approaches, MS‐based technologies such as GC‐MS, LC‐MS, and CE‐MS, NMR‐based strategy such as 2D NMR and solid‐state NMR, multidimensional chromatography, and MS hyphenated NMR techniques in addition to providing information on medicinal plants, where metabolomics efforts were demonstrated to be successful in identification of bioactive principles. The authors also demonstrate the utility of mass spectral imaging (MSI) and GNPS (Global Natural Products Social Molecular Networking) as emerging approaches in novel metabolite discovery processes.

Gupta et al^11^ provided an account of opportunities in conducting metabolomics studies to investigate prevalent neurological disorders in India. The authors stress that diseases such as acute spinal cord injury, amyotrophic lateral sclerosis, tethered cord syndrome, spina bifida, stroke, epilepsy, Parkinson's disease, and glioblastoma can be investigated using MS and NMR‐based approaches by allowing measurements of large numbers of metabolites in various disease pools that would allow quantification of “neurometabolites.”

In an exciting perspective, Phaple^12^ provided a unique dimension to the use of pharmaco‐metabolomics in drug development and clinical research in India. The author noticed that although for one and half decades the field has made advances in providing clinical evidence towards personalized medicine, the discovered pharmaco‐metabolomic biomarkers have not yet translated into the clinic due to a lack of large‐scale validation. Furthermore, the author calls on the Indian pharmaceutical research bodies to embrace the constantly improving bioanalytical infrastructure to embark on this opportunity.

In addition to thanking all the authors for their contributions to this special issue on metabolomics in India, we would like to thank all the reviewers, who have invested their time to help us peer‐review the manuscripts in a timely manner, and the journal's staff who have provided enormous logistics supports to help us pull this off together. As a first of its kind, this special issue will help motivate a generation of analytical scientists and metabolomicists in India, which will help move the field forward with greater impetus and vigor.

## CONFLICT OF INTEREST

The authors declare no financial interests/personal relationships, which may be considered as potential competing interests.
